# Brain Drain? PBDEs Alter Development of Human Brain Cells

**DOI:** 10.1289/ehp.118-a173a

**Published:** 2010-04

**Authors:** Kellyn S. Betts

**Affiliations:** **Kellyn S. Betts** has written about environmental contaminants, hazards, and technology for solving environmental problems for publications including *EHP* and *Environmental Science & Technology* for more than a dozen years

A new laboratory study demonstrating that polybrominated diphenyl ethers (PBDEs) can alter human fetal brain cells may explain at least in part the neurotoxicity recently documented in epidemiologic studies of young children exposed to PBDEs and previously shown in animal models **[*EHP*** **118:572–578; Schreiber et al.]**. The new study, the first to examine PBDE neurotoxicity in a human cell–based system, links the brain cell alterations to endocrine disruption.

A wealth of data demonstrates that babies can be exposed to significant amounts of PBDE flame retardants both in the womb and through breastfeeding. Although all 3 PBDE formulations—penta, octa, and deca—are banned in Europe, and the penta and octa formulations were discontinued in the United States (deca also is banned in some states), PBDEs may still be used in some new U.S. products and in wares manufactured elsewhere. They also are found in a wide variety of older plastic consumer goods that remain in use in many homes, businesses, and automobiles. The PBDEs are known to migrate into indoor dust, posing a particularly high exposure risk to infants and toddlers because of their characteristic hand-to-mouth behavior.

To investigate how PBDEs may impact the developing fetal brain, the team of scientists employed a method for evaluating human developmental neurotoxicity they had recently developed [*EHP* 117:1131–1138] as an alternative to animal testing. This method uses primary fetal human neural progenitor cells (hNPCs) cultured to produce complex 3-dimensional cellular systems called neurospheres. Neurospheres undergo the same basic processes that occur during the early stages of normal human brain development: cell proliferation, differentiation, and migration. Tests conducted with neurospheres may help identify exogenous substances that disturb these basic processes *in vivo*.

The researchers focused on 2 of the PBDE compounds that accumulate the most in humans, BDE-47 and BDE-99. At concentrations below levels that cause cell death, they found these compounds could reduce the migration of the hNPCs—which suggests the possibility of adverse effects on brain development—and the effects increased with higher PBDE concentrations. At the highest tested concentration (10 μM), BDE-47 decreased the distance the cells migrated by more than 25% compared with unexposed cells, whereas the same concentration of BDE-99 decreased the distance by more than 30%. Additional testing established that both compounds also interfered with the differentiation of immature progenitor cells into neurons and oligodendrocytes.

Further tests suggested the PBDE compounds affected cell migration and differentiation by interfering with thyroid hormone signaling, an endocrine-disrupting effect that could be associated with additional impacts throughout a person’s life. Followup work to determine whether PBDEs cause the same effects in rodent neurospheres would facilitate extrapolation from animals to humans, the authors say.

Other work has shown the tendency of PBDEs to accumulate in brain and neuronal cells, and the researchers used radiolabeled BDE-47 to measure an accumulation in test cells of approximately 60-fold. Considered with available human exposure data, these data suggest current PBDE exposure levels are likely to be of concern for human health, the authors write. Two recent studies have linked PBDEs with subtle changes in childrens’ IQs and behavior [*EHP* 117:1953–1958 and *EHP* doi:10.1289/ehp.0901340], and the authors say additional studies are needed to assess these associations.

## Figures and Tables

**Figure:**
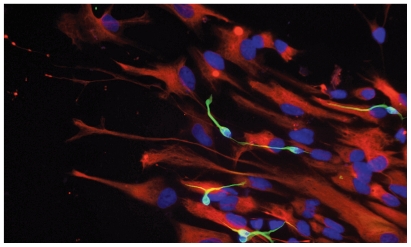
This fluorescence micrograph of differentiated neural progenitor cells shows neurons in green, glial cells in red, and cell nuclei in deep blue (the turquoise results from the overlay of the blue and green channels).

